# Severe Bilateral Lower Extremity Pyoderma Gangrenosum

**Published:** 2016-11-21

**Authors:** Jake Laun, Joshua B. Elston, Michael A. Harrington, Wyatt G. Payne

**Affiliations:** ^a^Division of Plastic Surgery, Department of Surgery, University of South Florida Morsani College of Medicine, Tampa; ^b^C.W. Bill Young Bay Pines VA Medical Center, Bay Pines, Fla

**Keywords:** pyoderma gangrenosum, pathergy, wound, lower extremities, TNF-α inhibitors

## DESCRIPTION

A 78-year-old man with a history of ulcerative colitis presented with progressive bilateral lower extremity wounds causing severe pain preventing ambulation. After excluding other possible diagnoses through various testing and biopsy, the presumptive diagnosis of pyoderma gangrenosum (PG) was suspected despite the patient's history of failing prednisone and infliximab therapy.

## QUESTIONS

1. **What is PG?**
2. **How does PG present?**
3. **How is PG diagnosed?**
4. **How is PG treated and what is the role of surgery in its management?**

## DISCUSSION

Pyoderma gangrenosum is a rare, poorly understood disease characterized by expansive ulcerating cutaneous lesions. Occurring in approximately 1:100,000, it is mainly observed in young to middle-aged individuals, with a female predominance; however, it may affect people of all ages.^1^,^2^ Pyoderma gangrenosum is associated with autoimmune disorders such as inflammatory bowel disease (IBD), rheumatoid arthritis, as well as neutrophilic dysregulation disorders such as Sweet's or Behcet's syndrome 50% to 70% of the time.^1^,^3^,^4^ Of note, PG can also appear as a paraneoplastic process in those with myeloproliferative malignancies.

**Figure 1:**
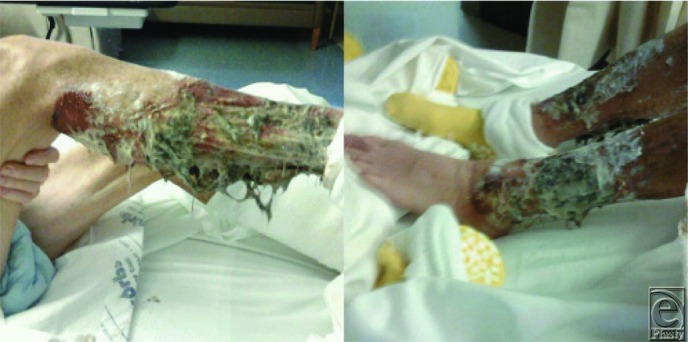
Severe bilateral lower extremity wounds that had progressed over months despite aggressive wound care and medical management with immunomodulators and steroids.

Pyoderma gangrenosum usually starts as a small, sterile inflammatory nodule or pustule at the site of minor trauma, which transforms into an ulcerative lesion.^3^ The most common location is in the pretibial region, but it may occur anywhere.^1^ Pyoderma gangrenosum may also be associated with systemic constitutional symptoms due to the elevation of IL-1B and the promotion of the inflammatory cascade.^1^ It can extend in a symmetric or asymmetric manner or by developing satellite lesions at sites of trauma (Fig 1).^3^ This process, known as pathergy, is one of the reasons why immune system dysregulation, specifically regarding neutrophils, is believed to be part of the pathophysiology.

Pyoderma gangrenosum is mainly diagnosed clinically, as there is currently no laboratory test specific to the disease and it is often a diagnosis of exclusion. Misdiagnosis is common due to its various presentations and ability to resemble other diseases such as infection, vasculitis, diabetes, or traumatic ulcers. The diagnostic workup should include a cutaneous biopsy, especially of the border of the ulcer. Malignancy and vasculitis can quickly be excluded, as PG has no specific diagnostic features on histopathology.^1^ Workup should include a rheumatologic panel including an antinuclear antibody and antineutrophil cytoplasmic antibodies to screen for IBD or autoimmune conditions, and a colonoscopy may be useful to evaluate for IBD.

**Figure 2:**
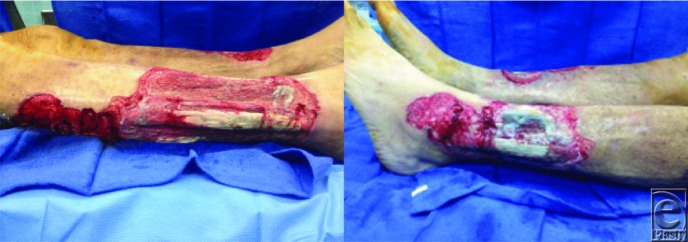
After conservative debridement of all necrotic material on the bilateral lower extremities. Multiple tendons are noted in the bilateral lateral compartments of the legs, with beefy-appearing proud tissue noted at the wound margins.

**Figure 3:**
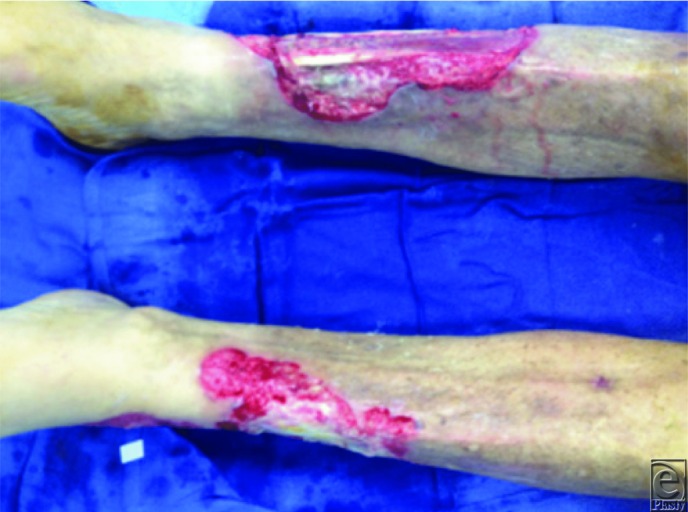
Straight down view demonstrating involvement of bilateral lower extremities with exposed tendons.

Treatment of any underlying pathology should be primary with the usage of corticosteroids, immunosuppressants, or TNF-α inhibitors. In some cases, this may address both the causative condition and the PG.^1^ No definitive local wound care regimen has been established because of varied local response; however, the goal is to maintain a moist environment to promote wound healing. Topical agents such as corticosteroids, tacrolimus, and cyclosporine are important, especially around the inflamed borders of the ulcer; however, they are highly absorbed and tacrolimus levels, especially, should be monitored.^5^ Topical antimicrobials such as mupirocin or silver sulfadiazine can be used to prevent superinfection. Petrolatum dressings may be used in the periwound area to prevent further skin irritation. Wet to dry or adherent dressings should absolutely be avoided, as they may aggravate the pathergy associated with PG.^5^-^7^ Because of the process of pathergy, surgical intervention is only indicated if the disease is controlled or profound necrosis is present (Figs 2 and 3). Skin grafting should only be attempted in patients currently undergoing immunosuppressive therapy, as ulcers may even develop at the skin graft donor site. Total colectomy may cause remission if IBD is the underlying disorder; however, recurrence may develop at the stoma. Ultimately, even with effective treatment, PG has a chronic and relapsing course.

Pyoderma gangrenosum can be a devastating disease of expanding cutaneous ulcerations clinically diagnosed once others are excluded. Clinical history can be useful if there is a known concomitant autoimmune disease. Treatment usually involves maintaining a moist environment conducive to wound healing with adjuvant systemic therapy to control the underlying disease process. Surgical debridement is reserved for grossly necrotic tissue or in the setting of controlled disease due to the inherent risk of pathergy.
